# Recent developments on the application of molecular probes in multiple myeloma: Beyond [^18^F]FDG

**DOI:** 10.3389/fbioe.2022.920882

**Published:** 2022-08-26

**Authors:** Shaojuan Zhang, Jingjie Shang, Weijian Ye, Tianming Zhao, Hao Xu, Hui Zeng, Lu Wang

**Affiliations:** ^1^ Center of Cyclotron and PET Radiopharmaceuticals, Department of Nuclear Medicine and PET/CT-MRI Center, The First Affiliated Hospital of Jinan University, Guangzhou, China; ^2^ Department of Hematology, The First Affiliated Hospital of Jinan University, Guangzhou, China

**Keywords:** multiple myeloma, biomarkers, molecular functional imaging, positron emission tomography (PET), radiotracers

## Abstract

Multiple myeloma (MM) is a neoplastic plasma cell proliferative disorder characterized by various osteolytic bone destruction as a radiological morphological marker. Functional imaging, particularly nuclear medicine imaging, is a promising method to visualize disease processes before the appearance of structural changes by targeting specific biomarkers related to metabolism ability, tumor microenvironment as well as neoplastic receptors. In addition, by targeting particular antigens with therapeutic antibodies, immuno-PET imaging can support the development of personalized theranostics. At present, various imaging agents have been prepared and evaluated in MM at preclinical and clinical levels. A summary overview of molecular functional imaging in MM is provided, and commonly used radiotracers are characterized.

## 1 Introduction

Multiple myeloma (MM) is caused by abnormal plasma cell infiltration in the bone marrow and is a final presentation of a range of monoclonal gammopathies, characterized by clinical symptomatic CRAB features including hypercalcemia, renal insufficiency, anemia, and bone lesions. In the light of the amount of clonal bone marrow plasma cells and serum monoclonal protein, monoclonal gammopathy of undetermined significance (MGUS) and smoldering multiple myeloma (SMM) are defined as its asymptomatic and premalignant stages (Rajkumar et al., 2014; Kumar et al., 2017). The potential risk of SMM (10% per year) (Kyle et al., 2007) and MGUS (1%–1.5% per year) for progression to symptomatic MM, emphasizing the importance of early monitoring and management initiation for high-risk patients (Kyle et al., 2010). On the other hand, MM is not only a highly heterogeneous disease but is also relapsing-remitting cancer, which means MM is treatable but incurable (Yang et al., 2020). Additionally, due to underlying molecular variation, the clinical disease course and optimal treatment or re-treatment strategy vary from person to person (Hideshima et al., 2007). Thus, early. Accurate assessment of residual MM-associated intramedullary and (or) extramedullary lesions is desirable for guiding further management. In 2016, the International Myeloma Working Group (IMWG) incorporated minimal residual disease (MRD) as a standard criterion in the evaluation of treatment response (Kumar et al., 2016). Recently, the utility of MRD negativity as an important prognostic marker for long-term survival in MM patients was confirmed by a large meta-analysis (Munshi et al., 2020).

New imaging techniques have come into being a part of the new Durie/Salmon PLUS staging system, considering anatomic and functional imaging for myeloma staging (Durie, 2006). Currently, modern recommended imaging technologies include whole-body low-dose computed tomography (WBLWCT), positron emission tomography/computed tomography (PET/CT), or whole-body magnetic resonance imaging (WB-MRI) (Mosebach et al., 2019; Terao and Matsue, 2022). A good detailed comparison of those imaging techniques have been reported by Zamagni, et al. (2019) In general, WBLDCT is a practical tool in the preliminary assessment of myeloma bone disease, considering its availability. For the differentiation between MGUS and SMM, which is warranted for serological and biopsy data, CT-guided biopsy is the gold standard (Mosebach et al., 2019). Combining with anatomical information from WBLWCT, PET/CT, imaged with radionuclides and WB-MRI tracked with hydrogen atom signal intensity with no radiation exposure, are recommended as reliable techniques for diagnostic workup and assessment and monitoring of therapy response in MM patients (Pawlyn et al., 2016; Ormond Filho et al., 2019). Due to the high spatial resolution of bone marrow, WB-MRI is highly recommended over [^18^F]fluorodeoxyglucose ([^18^F]FDG) PET/CT for the detection of the early and diffuse type of bone marrow infiltration, thus plays a key role in detecting small bone marrow infiltrations (∼5 mm) in the clinical diagnosis of suspected SMM patients (Dimopoulos et al., 2015), also helping re-identify MRD negativity (Zamagni et al., 2020). In particular, MRI functional approaches, like dynamic contrast-enhanced imaging (DCE) and diffusion weight imaging (DWI). As a functional alternative to WB-MRI, PET/CT with [^18^F]FDG can be used to depict contemporary lytic bone lesions along with glucose metabolism. More important, bone marrow signal in MRI scans (including DWI MRI) is greatly affected by individual age and treatment conditions and thus is suboptimal for early assessment of treatment response, but based on the ability of [^18^F]FDG PET/CT to distinguish between metabolically active and inactive diseases, as well as the “self-pop out” of avid lesions, which has great advantages in detecting extramedullary disease (EMD) and defines the imaging MRD-negative response to therapy (Cavo et al., 2017). Hybrid PET/MRI, it should be noted, is a promising “double” functional imaging technique, combining the advantages of MRI in the detection of bone marrow involvement and [^18^F]FDG PET in the prediction of both prognosis and treatment response (Mulé et al., 2020; Rama et al., 2022), systematic clinical data is required for proving the benefit of its sound added-value.

**FIGURE 1:**
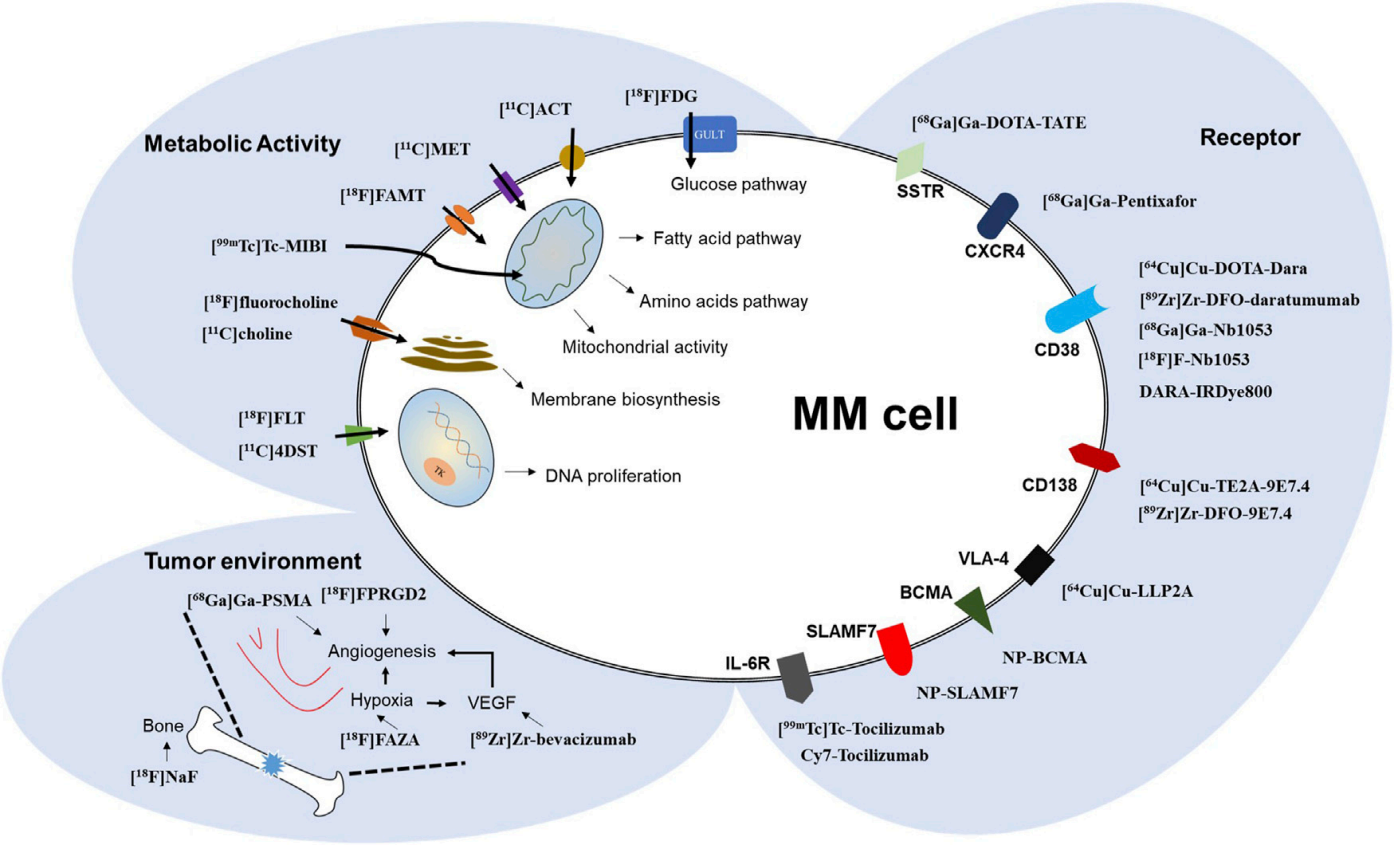
Schematic diagram of MM molecular imaging targets and associated imaging probes.

Functional imaging can objectively measure levels of pathogenic related-biomarker, making biomarker-targeted imaging a promising strategy to promote biologically personalized treatments for MM patients (Pawlyn and Davies, 2019), by enabling the identification of disease activity from different *in vivo* molecular perspectives such as metabolic activities, neoplastic microenvironment, and some specific receptors (Sachpekidis et al., 2019). Currently, [^18^F]FDG PET/CT in nuclear imaging, as the main type of functional imaging modalities, however, [^18^F]FDG is just an index reflecting glucose consumption, can’t help but wonder if there is a better imaging probes with better performance ability than [^18^F]FDG to assess and monitor MM lesions, especially, with increasing treatments with an immunotherapeutic agent by targeting specific receptors (Nadeem et al., 2020). This issue not only has spurred the use of “old” (originally mainly used for other tumors) imaging probes in MM, but inspired “new” imaging probes been developing for imaging MM (shown in Figure 1). (de Waal et al., 2017) Among these, immuno-PET which uses therapeutic antibodies to identify specific surface antigens has shown great promise in radio-immunotherapy and treatment monitoring, including detection of MRD (Pandit-Taskar, 2018). In perspective of different mechanisms of medical imaging, this review discusses the applications of variously reported imaging probes, mainly PET radiotracers, for their potential further use compared to [^18^F]FDG in MM.

## 2 Mechanism of medical imaging

### 2.1 Metabolic activity

#### 2.1.1 Glucose

[^18^F]FDG, a glucose analog, is subjected to the glucose metabolism pathway after intravenous injection. High retention of [^18^F]FDG is associated with active energy metabolism in myeloma cells and is related to increased numbers of glucose transporters (GLUT), mainly GLUT-1 and GLUT-3 (Pauwels et al., 1998). Subsequent phosphorylation of [^18^F]FDG by hexokinase makes it unable to escape cells or follow the glucose pathway, trapping it intracellularly (Kanazawa et al., 1986). To obtain optimal imaging qualities, some pre-scan patient preparations, including fasting, confirmation of normal blood glucose levels and a post-injection rest period, are required. In most clinical cases, PET is combined with CT to allow precise anatomical localization of the area of high tracer accumulation, and data is quantified as a standardized uptake value (SUV), traditionally SUVmax, SUVmean, and SUVratio (commonly indexed to liver values) (Huang, 2000). Under normal physiological states, homogeneous uptake of [^18^F]FDG in the bone marrow is low and less intense than that in the liver. A positive scan result with focal or diffuse active bone marrow uptake indicates that the disease is at an active and advanced stage, while a negative scan means a remission stage (van Lammeren-Venema et al., 2012). Evidence has shown that the number of abnormal avid lesions and associated changes in metabolic uptake after treatment are highly related to patient outcome, and can serve as an independent prognostic factor (Zamagni et al., 2016). For newly diagnosed MM patients receiving therapy, the uptake of bone lesions at levels lower than liver uptake can be thought of as a complete metabolic response, as referenced in the PET response criterion (shown in Table 1), (Zamagni et al., 2021) and is intertwined with MRD negativity.

**TABLE 1:** Proposed refinement of the PET response criteria after therapy.

PET response after therapy	Response criteria
complete metabolic response	Uptake ≤ liver activity in bone marrow sites and focal lesion(s) previously involved (including extra-medullary and para-medullary disease) (DS1–3)
partial metabolic response	Decrease in the number and/or activity of bone marrow/focal lesion(s) present at baseline, but persistence of lesion(s) with uptake > liver activity (DS 4 or 5)
stable metabolic disease	No significant change in bone marrow/focal lesion(s) compared to baseline
progressive metabolic disease	New focal lesion(s) compared to baseline consistent with myeloma

Many factors can cause false-positive or negative results, including 1) patients lacking the hexokinase enzyme (10%–15%) critical to trapping [^18^F]FDG in cells (Rasche et al., 2017); 2) changes in bone marrow uptake after therapy (e.g., recent chemotherapeutic drugs or use of cell growth factors) (Sugawara et al., 1998); 3) non-myeloma-associated high uptake (e.g., benign bone inflammation changes). Dynamic tracking can be combined with clinical patient information to reduce misinterpretation. However, more sensitive and specific imaging probes represent preferable alternative approaches for improving MM detection accuracy, which is complementary to the values of [^18^F]FDG imaging (shown in Tables 2, 3).

**TABLE 2:** Reported MM related imaging probe at clinical evaluation level.

Study index; Ref	Imaging probe	Clinical MM setting	Characteristic/Compared to [^18^F]FDG PET results
Ho CL 2014; Ho et al. (2014)	[^11^C]ACT	35 untreated patients (26 with MM, 5 with SMM, and 4 with MGUS)	1. MM patients: higher sensitivity (84.6% vs*.* 57.7%) and specificity (100% vs*.* 93.1%) compared to MRI
			2. SMM and MGUS patients: negative in [^11^C]ACT, but 2 MGUS positive in [^18^F]FDG
Lin C 2014; Lin et al. (2014)	[^11^C]ACT	15 MM patients	1. Diffuse lesions: higher detection rate (100% vs*.* 60%) with higher SUVmax (7.4 ± 3.9 vs*.* 3.3 ± 1.7)
			2. Focal lesions: higher detection ability (59 lesions vs*.* 29 lesions) with higher SUVmax (11.4 ± 3.3 vs*.* 6.6 ± 3.1)
			3. Response assessment: diffuse bone marrow uptake reduction of SUVmax (52% vs*.* 22%)
Nanni C 2007; Nanni et al. (2007)	[^11^C]choline	10 MM patients	bone lesions: comparable detection ability (37 lesions vs*.* 22 lesions) with higher SUVmax (5.0 vs*.* 3.8)
Cassou-Mounat T 2016; Cassou-Mounat et al. (2016)	[^18^F]fluorocholine	21 MM patients	bone lesions: higher detection ability (121–124 lesions vs*.* 69–71 lesions) with higher SUVmax (in 71%–73% lesions)
Meckova Z 2018; Meckova et al. (2018)	[^18^F]fluorocholine	5 MM patients	bone lesions: higher detection ability (134 lesions vs*.* 64 lesions) with comparable SUVmax (6.6 ± 1.6 vs*.* 6.5 ± 1.8)
Nakamoto Y 2013; Nakamoto et al. (2013)	[^11^C]MET	20 patients (15 with MM and 5 with plasmacytoma)	bone lesions: comparable detection ability (156 lesions vs*.* 58 lesions) with higher SUVmax (10.3 ± 5.6 vs*.* 3.4 ± 2.7)
Okasaki M 2015; Okasaki et al. (2015)	[^11^C]MET	46 patients with MM and 3 with MGUS (21 previously untreated, 43 restaged after treatment)	1. Bone lesions: higher detection ability (39 lesions vs*.* 33 lesions) with higher SUVmax 5.19 ± 2.40 vs*.* 3.35 ± 1.70)
			2. Lesion activity: higher sensitivity (86.7% *v*s. 60.0%) and equal specificity (76.1% vs*.* 76.1%) compared to marrow plasma cells cytology
Lapa C 2017; Lapa et al. (2017)	[^11^C]MET	78 patients (4 with solitary plasmacytoma, 5 with SMM and 69 with symptomatic MM)	1. MM patients: higher detection rate (75.6% vs*.* 60.3%)
			2. EMD: higher detection rate (72 foci vs*.* 44 foci)
			3. The first evidence of histologically proven [^18^F]FDG negative MM detectable by [^11^C]MET
Lapa C 2019; Lapa et al. (2019)	[^11^C]MET	19 patients (18 with MM and 1 with solitary bone plasmacytoma)	Bone lesions: higher detection rate in 42.1% patients of [^11^C]MET than [^11^C]choline
	[^11^C]choline		
Isoda A 2012; Isoda et al. (2012)	[^18^F]FAMT	11 MM patients (3 with newly diagnosed and 8 with relapsed)	bone lesions: comparable detection ability with lower SUVmax (2.0 ± 1.0 vs*.* 3.1 ± 1.2)
Luthra K 2014; Luthra et al. (2014)	[^99m^Tc]Tc-MIBI	84 patients (24 with newly diagnosed MM; 35 with treated MM, 2 with SMM, 4 cwith plasmacytoma, 13 with MGUS and 3 with suspected MM)	1. [^99m^Tc]Tc-MIBI uptake earlier than CT
			2. Follow-up patients: the presence or absence of [^99m^Tc]Tc-MIBI uptake could differentiate active from old burnt-out lesions
Mosci C 2020; Mosci et al. (2020)	[^99m^Tc]Tc-MIBI	62 newly diagnosed MM patients	1. Diffuse lesions: higher detection rate (78% vs*.* 58%)
			2. Focal lesions: lower detection rate (54% vs*.* 81%)
Sachpekidis C 2018; Sachpekidis et al. (2018)	[^18^F]FLT	8 myeloma patients (4 symptomatic MM and 4 with SMM)	bone lesions: lower detection ability (17 lesions vs. 48 lesions)
Okasaki M 2015; Okasaki et al. (2015)	[^11^C]4DST	46 patients with MM and 3 with MGUS (21 previously untreated, 43 restaged after treatment)	1. Bone lesions: higher detection ability (40 lesions vs. 33 lesions) with higher SUVmax (8.30 ± 6.24 vs. 3.35 ± 1.70)
			2. Lesion activity: higher sensitivity (93.3% vs. 60.0%) and lower specificity (71.4 % vs. 76.1%) compared to marrow plasma cells cytology
Withofs N 2017; Withofs et al. (2017)	[^18^F]FPRGD2	4 MM patients (2 with newly diagnosed and 2 with relapsed)	bone lesions: lower detection rate than WBCT (44 lesions vs*.* 80 lesions) and [^18^F]NaF/[^18^F]FDG PET/CT (44 lesions vs*.* 56 lesions)
de Waal EG 2015; de Waal et al. (2015)	[^18^F]FAZA	5 relapsed MM patients	bone lesions: negative scan
de Waal EG 2015; de Waal et al. (2017)	[^89^Zr]Zr-bevacizumab	5 relapsed MM patients	bone lesions: negative scan
Alabed YZ 2020; Alabed (2020)	[^68^Ga]Ga-PSMA	1 patient with multiple solitary plasmacytomas	bone lesions: positive scan
Dyrberg E 2017; Dyrberg et al. (2017)	[^18^F]NaF	14 patients newly diagnosed MM	1. Bone lesions: higher detection ability (41 lesions vs. 13 lesions)
			2. EMD: not recommended
Sonmezoglu K 2017; Sonmezoglu et al. (2017)	[^68^Ga]Ga-DOTA-TATE	19 MM patients	bone lesions: comparable detection ability (108 lesions vs*.* 112 lesions)
Pan Q 2020; Pan et al. (2020)	[^68^Ga]Ga-Pentixafor	30 patients with newly diagnosed MM	1. MM patients: higher detection rate (93.3% vs. 53.3%)
			2. Diffuse lesions: higher detection rate (88.2% vs*.* 29.4%) with higher SUVmax (7.8 ± 3.5 vs. 2.5 ± 0.9)
			3. Focal lesions: higher detection rate (92.3% vs*.* 69.2%) with higher SUVmax (20.4 ± 17.4 vs. 8.9 ± 5.6)
			4. [^68^Ga]Ga-Pentixafor uptake values related to tumor burden
Ulaner GA 2020; Ulaner et al. (2020)	[^89^Zr]Zr-DFO-daratumumab	10 MM patients	1. MM patients: 50% detection rate
			2. Identify lesions in one patient not seen at [^18^F]FDG PET/CT.

**TABLE 3:** Reported MM related imaging probe at pre-clinical evaluation level.

Study index; Ref	Imaging probe	Pre-clinical MM setting	Characteristic*/*Compared to [^18^F]FDG PET results
Soodgupta D 2016; Soodgupta et al. (2016)	[^64^Cu]Cu-LLP2A	5TGM1-GFP cells bearing mice	1. High specificity
			2. Comparable SUVmax with [^18^F]FDG
Bailly C 2019; Bailly et al. (2019)	[^64^Cu]Cu-TE2A-9E7.4	5T33-MM cells bearing mice	higher tumor to background ratio of [^64^Cu]Cu-TE2A-9E7.4 than [^89^Zr]Zr-DFO-9E7.4 at 24 h post-injection (4.08 ± 1.09 %ID/g vs*.* 1.42 ± 0.24 %ID/g)
	[^89^Zr]Zr-DFO-9E7.4		
Caserta E 2018; Caserta et al. (2018)	[^64^Cu]Cu-DOTA-Dara	MM.1S GFP^+^/Luc^+^ cells bearing mice	higher resolution and specificity than [^18^F]FDG
Ulaner GA 2020; Ulaner et al. (2020)	[^89^Zr]Zr-DFO-daratumumab	CD38^+^ OPM2 cells bearing mice	with and without blocking bone marrow uptake (5.4% ID/g vs*.* 16.2% ID/g)
Wang C 2021; Wang et al. (2021)	[^68^Ga]Ga-NOTA-Nb1053	MM.1S cells bearing mice	higher tumor to background ratio than [^18^F]FDG
Wei W 2021; Wei et al. (2021)	[^18^F]F-Nb1053	MM.1S cells bearing mice	high specificity through daratumumab premedication
Cho N 2021; Cho et al. (2021)	DARA-IRDye800	MM.1S GFP^+^/Luc^+^ cells bearing mice	1. High tumor to background ratio (5- and 18-fold)
			2. High specificity (11-fold decrease) after therapeutic doses of daratumumab
Detappe A 2019; Detappe et al. (2019)	NP-BCMA	MM.1S GFP^+^/Luc^+^ cells bearing mice	higher sensitivity and specificity of NP-BCMA than NP-SLAMF7
	NP-SLAMF7		
Ghai A 2021; Ghai et al. (2021)	[^89^Zr]Zr-DFO-elotuzumab	MM.1S cells bearing mice	higher sensitivity and specificity than [^18^F]FDG
Camacho X 2021; Camacho et al. (2021)	[^99m^Tc]Tc-Tocilizumab	MM.1S cells bearing mice	longer tumor uptake time of Cy7-Tocilizumab than [^99m^Tc]Tc-Tocilizumab
	Cy7-Tocilizumab		

#### 2.1.2 Fatty acid

Lipogenesis is a shared feature of a variety of malignant cells, and increased fatty acid synthase (FAS) expression has been observed in MM samples and human myeloma cell lines (Wang et al., 2008). [^11^C]acetate ([^11^C]ACT), an exogenous acetate, can be rapidly taken up by cells and metabolized to produce acetyl CoA, a carbon source for fatty acid synthesis. In a heterogeneous group of MM patients, [^11^C]ACT PET/CT exhibited better overall sensitivity and specificity than [^18^F]FDG. Furthermore, [^11^C]ACT PET/CT imaging, but not [^18^F]FDG imaging, was negative for indolent plasma cell neoplasms (SMM and MUGS) (Ho et al., 2014). A similar study also showed that, for newly diagnosed MM patients, [^11^C]ACT imaging has a higher detection rate for a focal or infiltrated myeloma lesions than [^18^F]FDG. Moreover, this positive treatment response was visualized via [^11^C]ACT PET/CT as a significant decrease in SUVmax (listed in Table 2). (Lin et al., 2014)

#### 2.1.3 Membrane biosynthesis

Choline, which can be phosphorylated by choline kinase into phosphatidylcholine, is involved in cell membrane biosynthesis. The use of [^11^C]choline for MM patients can be justified based on the increase in proliferating malignant plasma cells with high demands related to membrane metabolism and growth. For MM patients, [^11^C]choline imaging was performed similarly to [^18^F]FDG imaging for lesion detection, treatment evaluation, and monitoring (Nanni et al., 2007). Subsequent PET imaging studies of choline were performed with labeled fluorine-18. In relapsing MM patients, [^18^F]fluorocholine PET/CT indicated that a significantly higher number of lesions were detected compared to [^18^F]FDG (Cassou-Mounat et al., 2016). Another study that applied [^18^F]fluorocholine to the detection of skeletal involvement showed that [^18^F]fluorocholine PET/CT detected about twice as many bone lesions as [^18^F]FDG, particularly on the skull bone (listed in Table 2). (Meckova et al., 2018) These results are likely due to higher background metabolic level noise for [^18^F]FDG.

#### 2.1.4 Amino acids

Amino acids are important substrates in the biosynthesis of lipid and protein molecules. [^11^C]methionine ([^11^C]MET) possesses a higher specificity than [^18^F]FDG for the detection of original and recurrent brain tumors and, due to its low physiological background, can also delimit surgical boundaries (Ogawa et al., 1987). Extensive MET is required for the unrestricted proliferation of plasma cells, along with the excessive synthesis of monoclonal immunoglobulins. Cellular transport of [^11^C]MET is determined by the sodium-independent L-type amino acid transporter (LAT). The physiological uptake of [^11^C]MET is distributed in the bone marrow and liver. High expression of LAT1 has been identified as a relevant prognostic factor associated with overall poor long-term survival (Isoda et al., 2014). Regarding patients, a study showed that more abnormal lesions were identified by [^11^C]MET PET/CT than by [^18^F]FDG, making it useful in grading disease stage (Nakamoto et al., 2013). Another study indicated that [^11^C]MET PET/CT detected a greater number of positive uptake lesions with more clarity than [^18^F]FDG, especially with 10–30% plasma cells in the bone marrow (Okasaki et al., 2015). In a study of 78 patients, the largest so far, [^11^C]MET PET/CT was shown to have higher sensitivity than [^18^F]FDG in detecting myeloma infiltrated lesions within or outside of bone marrow, as confirmed by histological biopsy. [^11^C]MET can be potentially applied to disease staging and re-staging with higher accuracy than [^18^F]FDG (Lapa et al., 2017). In addition, the same group also compared [^11^C]MET to [^11^C]choline, and the advantages of [^11^C]MET were supported by a higher detection rate of MM bone lesions in approximately 40% of patients, as well as higher SUVmax (listed in Table 2). (Lapa et al., 2019)

^18^F α-methyl tyrosine ([^18^F]FAMT), a fluorine-18 labeled version of the unnatural amino acid methyltyrosine, can also be transported into cells through LAT-1. Likewise, the uptake of [^18^F]FAMT by lesions is positively correlated with the expression of LAT-1. In MM patients, a comparable detection rate was observed for [^18^F]FAMT and [^18^F]FDG imaging, but uptake discrepancies were evident in several presented lesions (listed in Table 2). (Isoda et al., 2012)

#### 2.1.5 Mitochondrial activity

Technetium 99 m sestamibi ([^99m^Tc]Tc-MIBI) is a typical radiotracer used in single-photon emission computed tomography (SPECT) to investigate myocardial perfusion (Alexander and Oberhausen, 1995). Generally, the spatial resolution of SPECT is much lower than that of PET. The high lipid solubility of [^99m^Tc]Tc-MIBI allows it to enter the mitochondria along with the negative membrane potential difference formed by membrane electrophysiological activities. The lesion concentration reflects the high energy metabolism levels found within active malignant plasma cells. A study with 112 [^99m^Tc]Tc-MIBI SPECT was performed in 84 myeloma-associated patients, scan results indicated that the concentration of MIBI in myeloma lesions, corresponds with unchanged and changed radiological changes in CT, could expose earlier ongoing disease activity or old treated lesions (Luthra et al., 2014). In newly diagnosed MM patients, diffuse involvement of bone marrow was better visualized by [^99m^Tc]Tc-MIBI SPECT scan than by [^18^F]FDG PET/CT scan but was less efficient for focal lesions (listed in Table 2). (Mosci et al., 2020) [^99m^Tc]Tc-MIBI seems to be particularly useful in evaluating the existence of extensive infiltration to avoid underestimation of disease status, meanwhile, the low spatial resolution of SPECT limits the identification of small lesions.

#### 2.1.6 DNA proliferation

Pyrimidine 3-deoxy-3- (Mulé et al., 2020)F-fluorothymidine ([^18^F]FLT) and the newer tracer 4′-methyl- (Terao and Matsue, 2022)C-thiothymidine ([^11^C]4DST) can participate in DNA synthesis as thymidine analogs and have been used to image high DNA proliferation activity in cells (Toyohara et al., 2011; Peck et al., 2015). After being phosphorylated by thymidine kinase 1 (TK1), both compounds become metabolically trapped within cells. Due to the structures, [^11^C]4DST is more stable than [^18^F]FLT, and de-phosphorylation occurs relatively rarely. Like [^11^C]MET, [^11^C]4DST PET/CT can detect more bone lesions per patient than [^18^F]FDG in patients with low levels of plasma cell infiltration (10–30%) (Okasaki et al., 2015). However, [^11^C]4DST also tends to accumulate in active hematopoietic marrow, and has to be associated with the patient background to distinguish from diffuse MM lesions. In contrast, preliminary data indicate that [^18^F]FLT is not suitable for initial MM diagnostics due to the interference of background bone marrow activity in the cell compartment (Sachpekidis et al., 2018). Otherwise, [^18^F]FLT can be used to obtain updated information on the distribution of normal bone marrow tissue during therapy (listed in Table 2). (Hayman et al., 2011)

### 2.2 Tumor microenvironment

#### 2.2.1 Angiogenesis & hypoxia

Oxygen consumption is increased with the proliferation of malignant MM cells, resulting in a relatively hypoxic cellular environment that ultimately activates the vascular endothelial growth factor (VEGF) signaling pathway (Apte et al., 2019), leading to tumor angiogenesis. ^18^F-FB-NH-mini-PEG-E [c (RGDyK)]_2_ ([^18^F]FPRGD2) is a standard PET tracer for imaging integrin *α*_v_ *β*_3_, a type of integrin highly expressed by vascular endothelial cells, and can be used to image tumor angiogenesis (Wu et al., 2007). However, in relapsed MM patients, [^18^F]FPRGD2 PET/CT is not particularly helpful and was dependent on the presence of obvious lytic bone lesions found by CT (listed in Table 2). (Withofs et al., 2017)

1-α-D:-(5-deoxy-5-^18^F-fluoroarabinofuranosyl)-2-nitroimidazole ([^18^F]FAZA) is a PET tracer used to identify hypoxic conditions associated with tumor metabolism (Wuest and Wuest, 2013). When cells are oxygen-deficient, nitroimidazole reduction products will bind to intracellular biomacromolecules and remain in cells. One report indicated that no increased uptake of [^18^F]FAZA was found for any of five relapsing MM patients, while numerous focal uptakes presented on [^18^F]FDG PET/CT (de Waal et al., 2015). Likely due to the hypoxic nature of the whole bone marrow compartment, no differences were observed between MM lesions and their surroundings. Bevacizumab, the first humanized nanoantibody (mAb) approved by the Food and Drug Administration (FDA) to inhibit tumor angiogenesis, targets the VEGF receptor. Unfortunately, like [^18^F]FAZA, PET imaging with zirconium-89 labeled bevacizumab failed to detect significant abnormalities in all patients (listed in Table 2). (de Waal et al., 2017)

Prostate-specific membrane antigen (PSMA) is a characteristic biomarker for prostate cancer cells (Wester and Schottelius, 2019), and enhanced expression has also been observed in tumor vasculature. A case report indicated that ^68^Ga-prostate-specific membrane antigen-targeted ligand PET imaging can be used to visualize multiple lytic bone lesions throughout the spine (listed in Table 2), (Alabed, 2020) but the definite application in MM is still unclear.

#### 2.2.2 Osteoclastic lesions

Osteoclastic lesions result from increased plasma cell infiltration in the bone marrow microenvironment, which stimulates bone resorption and impedes bone formation (Mukkamalla and Malipeddi, 2021). The PET tracer [^18^F]NaF is ‘bone-depositing’, reflecting bone osteoblastic reactions related to regional blood flow. Thus, the typical accumulation of [^18^F]NaF around lesions can be explained by a secondary osteoblastic reaction, indicating that [^18^F]NaF is suitable for comprehensive evaluation of bone injury in late stages (listed in Table 2). (Dyrberg et al., 2017) And, [^18^F]NaF cannot detect EMD logically.

### 2.3 Receptor targeted imaging

#### 2.3.1 Somatostatin receptors

Somatostatin receptor scintigraphy (SRS) using [^111m^In]In-pentetreotide has been applied in the workup of neuroendocrine tumors (NETs) for visualizing somatostatin receptors (SSTR), particularly subtypes 2 and 5. Due to the advantages of PET regarding spatial resolution, ^68^Ga-tetraazacyclododecane-tetraacetic acid-octreotate ([^68^Ga]Ga-DOTA-TATE) PET/CT has largely replaced SRS for staging NET (Ambrosini et al., 2010). *In vitro* studies have shown that functional SST is expressed by all MM cell lines, predominantly SSTR5 (Georgii-Hemming et al., 1999). No significant difference was observed between [^18^F]FDG PET/CT, but diffuse bone marrow uptake can be better shown with [^68^Ga]Ga-DOTA-TATE (listed in Table 2). (Sonmezoglu et al., 2017)

#### 2.3.2 Very late antigen-4

Very late antigen-4 (VLA-4), a transmembrane adhesion receptor expressed on normal plasma cells, is an important contributor to interactions between plasma cells and the extracellular matrix and bone marrow stromal cells (Schlesinger and Bendas, 2015). Upregulated expression of VLA-4 has been confirmed for myeloma cells and surrounding tissues. With a high binding affinity, N-[[4-[[[(2-ethylphenyl)amino]carbonyl]amino]phenyl]acetyl]-N (epsilon)-6-[(2E)-1-oxo-3-(3-pyridinyl-2-propenyl)]-L-lysyl-L-2-aminohexanedioyl-(1-amino-1-cyclohexane)carboxamide (LLP2A) is a peptidomimetic ligand for VLA-4. LLP2A was conjugated to 1,4,8,11-tetraazacyclotetradecane-1-(methane phosphonic acid)-8-(methane carboxylic acid) (CB-TE1A1P) chelators for cuprum-64 labelling. Favorable pre-clinical results regarding the biodistribution and dosimetry of [^64^Cu]Cu-LLP2A imaging have been reported for MM mice models, suggesting that this approach is a promising candidate for further imaging of activated VLA-4 in humans (listed in Table 3). (Soodgupta et al., 2016)

#### 2.3.3 Chemokine receptor-4

A member of the G-protein-coupled chemokine receptor family, chemokine receptor-4 (CXCR4) is mainly expressed in bone marrow primitive hematopoietic cells and is involved in the survival of myeloma cells (Philipp-Abbrederis et al., 2015). Plerixafor (Wang et al., 2020), an exogenous CXCR4 antagonist with high binding affinity, can disrupt adhesive tumor-stroma interactions and achieve treatment goals. [^68^Ga]Ga-pentixafor PET/CT has been proposed as a theranostics tracer targeting CXCR4 for directed radio-targeted treatment with [^177^Lu]Lu-pentixafor (or [^90^Y]Y-pentixafor). In newly diagnosed MM patients, [^68^Ga]Ga-pentixafor exhibited superior detection ability for myeloma lesions compared to [^18^F]FDG (93.3% vs. 53.3%). What’s more, quantitative analysis results indicated that the uptake of [^68^Ga]Ga-pentixafor in bone marrow is a promising biomarker for tumor burden assessment, as it is positively correlated with serum β2-microglobulin and other clinical tumor burden parameters (Pan et al., 2020). A profound therapeutic impact was observed on two heavily pretreated patients following CXCR4-directed lutetium-177 or yttrium-90 endo-radiotherapy, with patients exhibiting a remarkable [^18^F]FDG uptake reduction in intra and extra-medullary lesions despite the ultimately limited 3–6 months progression-free survival (listed in Table 2). (Herrmann et al., 2016)

#### 2.3.4 Cluster of differentiation 138 and 38

Cluster of differentiation (CD) 138, or syndecan-1, is a type of transmembrane proteoglycan found at high levels on the surface of myeloma cells (Sanderson and Yang, 2008). It has been used as a positive sorting marker in the preconcentration of plasma cells for efficient cytogenetic analysis of bone marrow samples. In this respect, CD138 may be an important and potentially beneficial target for imaging and mAbs-based immunotherapy. Cuprum-64 or zirconium-89 labeled anti-CD138 antibodies were realized by incorporating 1,4,8,11-tetraazabicyclo [6.6.2]hexadecane (TE2A) or defetoxamine (DFO) chelator into the antibodies, thus delivering the immuno-PET tracers [^64^Cu]Cu-TE2A-9E7.4 and [^89^Zr]Zr-DFO-9E7.4, respectively. In the bone lesions of MM-bearing mice, higher uptake was observed during both PET imaging for [^64^Cu]Cu-TE2A-9E7.4 and [^89^Zr]Zr-DFO-9E7.44, but not for [^18^F]FDG. In addition, the osteophilicity of zirconium-89 resulted in undesired bone background. The higher signal-to-noise ratio of [^64^Cu]Cu-TE2A-9E7.4 indicated a potential use as a new specific option for MM imaging diagnosis (listed in Table 3). (Bailly et al., 2019) What’s more, mouse models and dosimetry results corroborated the feasibility of radioimmunotherapy for the treatment of advanced-stage MM using anti-CD138 monoclonal antibody namely B-B4, which was radiolabeled with bismuth-213 to promote longer median survival (Chérel et al., 2013).

Daratumumab (Dara), a humanized IgG1K mAb approved by the FDA for use in relapsed MM (Lokhorst et al., 2015), is targeted to the receptor cluster of differentiation 38 (CD38) upregulated in malignant plasma cells but remained low-level expression by surrounding hematopoietic cells. Therefore, CD38-targeted imaging offers a novel approach to the dynamic and invasive assessment of its expression in MM. In preclinical studies, [^64^Cu]Cu-DOTA-Dara displayed satisfactory potency for CD38 imaging on the surface of MM cell lines, primarily associated with bone infiltration foci. The higher specificity of [^64^Cu]Cu-DOTA-Dara PET/CT compared to [^18^F]FDG supports possible clinical applications for MRD detection (listed in Table 3). (Caserta et al., 2018) Recently, zirconium-89 labeled CD38-targeting antibodies for MM were generated using the chelator DFO, providing the first published proof-of-principle for chemical synthesis, preclinical evaluation, and Phase 0 imaging in humans (Ulaner et al., 2020). The results showed that [^89^Zr]Zr-DFO-daratumumab has a robust ability to visualize CD38 in the murine model (listed in Table 3), and the use of this immuno-PET tracer could be a valuable diagnostic approach due to its high specificity. In addition, full antibody imaging means that a single injection can be sufficient for four PET/CT scans on different days. In humans, the dosimetry of [^89^Zr]Zr-DFO-daratumumab was found to be acceptable and within safe limits. The Phase 0 clinical trials by [^89^Zr]Zr-DFO-daratumumab PET/CT imaging study included 10 MM patients and half of them demonstrated avidity on osseous lesions, especially one patient who demonstrated unexpected focal tracer uptake previously undetected by ^18^F-FDG, consistent with the lack of uptake in low CD38 expression lesions demonstrated by molecular detection approaches (listed in Table 2). By labelling a CD38-specific nanobody (Nb1053) with gallium-68 (Wang et al., 2021) or fluorine-18 (Wei et al., 2021), two preclinical studies indicated that Nb1053-based molecular imaging radiotracers may be useful for MM diagnosis and follow-up (listed in Table 3). Another imaging technique is NIR fluorescence imaging. Preclinical evaluation of DARA-IRDye800, in which DARA conjugated to the NIR fluorophore IRDye800CW, revealed a significant (∼10×) reduction *in vivo* in fluorescence intensity for the treated group (listed in Table 3). (Cho et al., 2021)

#### 2.3.5 B cell maturation antigen and signaling lymphocyte activation molecule 7

B cell maturation antigen (BCMA) is a member of the tumor necrosis factor receptor superfamily that is found almost exclusively on mature B cells. Its expression level increases significantly in MM cells, and its expression level is positively correlated with MM progression (Shah et al., 2020). In addition, signaling lymphocyte activation molecule 7 (SLAMF 7), which is expressed on immune cells including plasma cells, is a receptor involved in regulating MM cell migration within bone marrow stroma (Malaer and Mathew, 2017). By combining ultra-small sub-5 nm gadolinium-containing nanoparticles (NP) with BCMA and SLAMF 7 targeted antibodies, NP-BCMA and NP-SLAMF7 MR probes were successfully generated. Whole-body imaging of MM tumor-bearing mice showed that BCMA not only had better specificity than SLAMF 7 but also supported clearer imaging of lesions (listed in Table 3). (Detappe et al., 2019)

In terms of radiotracers, elotuzumab, a human monoclonal antibody against SLAMF7 that has been approved by the FDA for use in relapsed MM, has been labeled with zirconium-89. Micro-PET imaging with [^89^Zr]Zr-DFO-elotuzumab in MM tumor mice indicated that it can specifically identify bone lesions with high expression of SLAMF7. SUVmax was significantly higher than that of [^18^F]FDG, suggesting that [^89^Zr]Zr-DFO-elotuzumab can be used to evaluate changes in tumor load after elotuzumab treatment (listed in Table 3). (Ghai et al., 2021) Regarding immunotherapy, BCMA would be a better choice due to its exclusively high expression in malignant plasma cells. Remarkable clinical effects have been witnessed in patients with relapsed/refractory multiple myeloma (RRMM) following antibody-drug conjugate (ADC) treatment, a type of BCMA-targeted therapeutics (Demel et al., 2021). Thus, further incorporation of PET radioisotopes with antibodies targeted to BCMA may be pursued to enhance sensitivity.

#### 2.3.6 Interleukin-6 receptor

Interleukin-6 (IL-6) is a cytokine with broad functions in inflammation and immunity that has been identified as a proliferative factor for MM (Zhang et al., 1992). The results of an early preclinical imaging study using technetium-99 m labeled or fluorophore Cy7-labeled tocilizumab, a humanized Ab that binds to the IL-6 receptor, showed that both [^99m^Tc]Tc-tocilizumab and Cy7-tocilizumab require a long time for uptake into MM engrafted tumors, with up to 72 h required for Cy7-tocilizumab (listed in Table 3), (Camacho et al., 2021) thus hampering further clinical translational application.

## 3 Conclusion

[^18^F]FDG, one of the most common medical probes used in MM functional imaging, has provided valuable guidance for the management of MM patients, like standard WB-MRI. Standardized clinical care and proper imaging evaluation criteria have been promoted for wide distribution. Due to the limitations of [^18^F]FDG in imaging MM, various probes, especially the radioisotope labeled PET tracers, have been suggested and assessed in clinical patients with related malignancies or during preclinical evaluation. Different imaging agents were used to identify various pathological features of MM; while their values are worth consideration, the primary pursuit in this review is superior performance compared to [^18^F]FDG (Table 2). Some of these agents, including metabolic tracers such as [^11^C]ACT, [^11^C]MET, and [^11^C]choline, have exhibited promising results in the detection of lesions in MM patients, and tend to have higher SUVmax than [^18^F]FDG. Based on the data reported so far, it may be suggested that metabolic characterization of lipid and protein metabolism can be more accurate than glucose metabolism in the early diagnosis, disease staging, and treatment response monitoring of MM. Meanwhile, by its short half-life (*t*_1/2_ = 20.4 min), carbon-11 makes a complementary PET/CT scan with [^18^F]FDG on the same day realizable. But their use is somewhat limited by the requirement for an on-site cyclotron and the only very few nuclear medicine centers so far. For [^11^C]choline, fluorine-18 (*t*_1/2_ = 109.8 min) labeled choline ([^18^F]fluorocholine) could be a good alternative. In terms of the background metabolic level noise, some metabolic PET tracers, such as [^18^F]FLT, [^18^F]fluorocholine, and [^11^C]choline, has unfavorable physiological distribution, characterized by increased uptake in the bone marrow and liver. Further validation of these agents in larger patient cohorts and clinical trials is important. Limited performance of tracers related to the tumor environment is also reflected in the workups of MM patients, and these agents do not appear to be individually useful for clinical evaluation, except [^99m^Tc]Tc-MIBI. [^99m^Tc]Tc-MIBI can be a good alternative for [^18^F]FDG PET/CT scan with a much lower cost, especially for late-stage MM patients.

Increasingly, MRD assessment has become a critical standard in the clinical assessment of MM, with major efforts to develop methods with sensitive detection and specific exclusion. The traditional treatment of MM has been revolutionized by the progression of immunotherapy. Meanwhile, immuno-PET imaging with radiolabeled antibodies or antibody fragments has potential for MRD assessment and optimization of personalized therapy, [^64^Cu]Cu-DOTA-Dara might helpful. In the context of theragnostic approaches to MM, the major advantage of the PET tracer [^68^Ga]Ga-pentixafor is its potential for use in combination with the therapeutic lutetium-177 or yttrium-90 labeled pentixafor in progressive MM patients with CXCR4-positive tumor cells, as confirmed by a [^68^Ga]Ga-pentixafor PET scan. Likewise, [^89^Zr]Zr-DFO-daratumumab could be used to identify MM patients who would benefit from daratumumab and thus predict the effectiveness of treatment. Additional research is needed to validate and explore the practical application indications of these novel agents in various MM clinical conditions. Most other reported probes are in very early preclinical development, but some agents, particularly the NP-BCMA have shown promising potential for further prospective studies, which also signifies the possibility and feasibility of a PET tracer for BCMA aimed at immuno-PET imaging. And zirconium-89 (*t*_1/2_ = 78.4 h) and cuprum-64 (*t*_1/2_ = 12.7 h) are the most common radioisotopes for antibody labelling. The “bone-seeking” nature of zirconium-89 must be considered to understand the intrinsic impact of immuno-PET imaging. Even though routine clinical use of immune-PET imaging is hindered by a lack of proper long-lived radionuclides and the availability of antibodies or corresponding fragments, mAb-based immune-PET holds the potential to maximize patient benefits through MRD detection and the promotion of immunotherapy.

## Glossary

[^11^C]4DST: 4′-methyl-^11^C-thiothymidine
[^11^C]ACT: [^11^C]acetate
[^11^C]MET: [^11^C]methionine
[^18^F]FAMT: ^18^F α-methyl-tyrosine
[^18^F]FAZA: 1-α-D: -(5-deoxy-5-^18^F-fluoroarabinofuranosyl)-2-nitroimidazole
[^18^F]FDG: [^18^F]fluorodeoxyglucose
[^18^F]FLT: Pyrimidine 3-deoxy-3-^18^F-fluorothymidine
[^18^F]FPRGD2: ^18^F-FB-NH-mini-PEG-E[c(RGDyK)]_2_
[^64^Cu]Cu-LLP2A: ^64^Cu-CB-TE1A1P-PEG4-LLP2A
[^68^Ga]Ga-DOTA-TATE: ^68^Ga-tetraazacyclododecane-tetraacetic acid-octreotate
[^99m^Tc]Tc-MIBI: Technetium 99 m sestamibi
ADC: antibody-drug conjugate
BCMA: B cell maturation antigen
CB-TE1A1P: 1,4,8,11-tetraazacyclotetradecane-1-[methane phosphonic acid]-8-[methane carboxylic acid]
CD: cluster of differentiation
CXCR4: chemokine receptor-4
Dara: Daratumumab
DCE: dynamic contrast-enhanced
DFO: Defetoxamine
DWI: diffusion weight imaging
FAS: fatty acid synthase
FDA: Food and Drug Administration
GLUT: glucose transporters
IL-6: Interleukin-6
IMWG: International Myeloma Working Group
LAT: L-type amino acid transporter
LLP2A: N-[[4-[[[(2-ethylphenyl)amino]carbonyl]amino]phenyl]acetyl]-N(epsilon)-6-[(2E)-1-oxo-3-(3-pyridinyl-2-propenyl)]-L-lysyl-L-2-aminohexanedioyl-(1-amino-1-cyclohexane)carboxamide
mAb: humanized nanoantibody
MGUS: monoclonal gammopathy of undetermined significance
MM: multiple myeloma
MRD: minimal residual disease
NETs: neuroendocrine tumors
NIR: near-infrared
NOTA: 1,4,7-triazacyclononane-N,N′,N″-triacetic acid
NP: nanoparticles
PET/CT: positron emission tomography/computed tomography
PSMA: prostate-specific membrane antigen
RRMM: relapsed/refractory multiple myeloma
SLAMF 7: signaling lymphocyte activation molecule 7
SMM: smoldering multiple myeloma
SRS: somatostatin receptor scintigraphy
SSTR: somatostatin receptors
SUV: standardized uptake value
TE2A: 1,4,8,11-tetraazabicyclo[6.6.2]hexadecane
TK1: thymidine kinase 1
VEGF: vascular endothelial growth factor
VLA-4: very late antigen-4
WBLWCT: whole-body low-dose computed tomography
WB-MRI: whole-body magnetic resonance imaging
EMD: extramedullary disease
